# Editorial: Advances in extracorporeal life support in critically ill patients, volume III

**DOI:** 10.3389/fmed.2024.1394830

**Published:** 2024-03-26

**Authors:** Guo-wei Tu, Nikola Dobrilovic, Man Huang, Zhe Luo

**Affiliations:** ^1^Cardiac Intensive Care Center, Zhongshan Hospital, Fudan University, Shanghai, China; ^2^Division of Cardiac Surgery, NorthShore University HealthSystem, Chicago, IL, United States; ^3^Department of Intensive Care Unit, Second Affiliated Hospital, School of Medicine, Zhejiang University, Hangzhou, China; ^4^Shanghai Key Laboratory of Pulmonary Inflammation and Injury, Shanghai, China

**Keywords:** extracorporeal membrane oxygenation, cardiogenic shock, acute respiratory distress syndrome, mobilization, pulsatility, hemorrhage

Extracorporeal life support (ECLS), also known as extracorporeal membrane oxygenation (ECMO), has emerged as a vital technology in critical care medicine. This advanced intervention provides temporary life support for patients with severe respiratory and/or cardiac failure, who are otherwise at high risk of mortality (1, 2). Over the years, ECLS has witnessed significant advancements, making it an indispensable tool in managing critically ill patients. Expanding its clinical applications have significantly improved patient outcomes, offering a glimmer of hope for those facing life-threatening conditions. As research and development continue to push the boundaries of ECLS, we can anticipate more groundbreaking innovations that will further elucidate its critical role in intensive care. In this editorial, we will explore the recent progress and potential future developments in the field of ECLS, shedding light on its profound impact on patient outcomes and the evolving role it plays in patient care.

In this Research Topic: *Advances in extracorporeal life support in critically ill patients, volume III* (https://www.frontiersin.org/research-topics/53764/advances-in-extracorporeal-life-support-in-critically-ill-patients-volume-iii), five articles were included, with three original research, one review, and one case report. They have offered the latest insights across a wide range of Research Topics, such as patient selection, ventilation strategy and mobilization.

Mobilization during venovenous extracorporeal membrane oxygenation (VV-ECMO) refers to the process of initiating and implementing physical activity for patients receiving ECMO support. It has gained increasing attention as early mobilization plays a crucial role in preventing complications associated with immobility and promoting enhanced recovery. Mobilization interventions during VV-ECMO include early and progressive ambulation, range of motion exercises, and strength training (3). These initiatives aim to improve cardiovascular function, respiratory mechanics, and overall functional status. Unfortunately, mobilization of ECMO patients is still not widely carried out and a lack of standardized protocol means the benefits of it remain to be explored. Rottmann et al. reported a single center experience in which different methods of mobilization was implemented. They found, compared to “in-bed” mobilization, active mobilization, such as being mobilized to a chair or standing, during VV-ECMO support was associated with improved 30-day survival and a higher rate of successful weaning from mechanical ventilation. This finding suggests that the potential benefits of mobilization may be protocol-dependent, and a more intense plan during ECMO support may be required to achieve its goal.

Ultra-protective ventilation (UPV), which incorporates extra lower tidal volumes, driving pressure, and respiratory rate, is an advanced ventilation strategy aiming to reduce the risk of ventilation-induced lung injury. Emerged as a novel idea in the late 80's, it has shown to be a safe option in human study and effectively reduce lung injury in animal models on VV-ECMO support. However, whether these strategies reduce lung inflammation more effectively than protective ventilation (PV) remains unclear. Deniel et al. conducted an experimental study on animals to evaluate the effect of UPV, and compared it with PV on acute lung macrophagic inflammation using advanced pathological methods. They found a combination of ultra-low tidal volume, high positive end expiratory pressure, low respiratory rate, and controlled plateau pressure reduced global lung inflammation compared to conventional PV. Such an exciting pre-clinical result adds more evidence to the possible clinical application of UPV in patients with VV-ECMO support.

The choice of ECLS system is a new focus in ECLS research as a number of modern systems have been developed. Currently, classical extracorporeal circulation systems are related to hemotologic complications and heightened inflammation (4, 5). Zieger et al. adopted a new system with pulsatile blood flow and examined its effects *ex-vivo*. They demonstrated that in the presence of pulsatility, neither reduced levels of inflammation nor fewer complications occurred. On the contrary, prolonged duration might lead to more severe hemolysis. These results should be considered carefully as the addition of pulsatility might be harmful.

In patients with severe asthma exacerbations, traditional mechanical ventilation may not provide sufficient support, leading to worsening respiratory failure. Several studies have reported positive outcomes with the use of VV-ECMO in asthma patients as a rescue therapy. Despite these advancements, complications occur frequently in this population group. Mechanical failure, bleeding, neurologic incidents and renal impairment occurred more frequently in non-survivors. Wang et al. reported a patient with severe asthma exacerbation who developed severe hypokalemia and fatal hemorrhage following VV-ECMO. Clinicians should carefully select patients and balance the potential risks and complications associated with ECMO therapy. Further research and refinement of protocol guidelines are necessary to optimize patient selection and improve outcomes in this challenging population.

Veno-Arterial Extracorporeal Membrane Oxygenation (VA-ECMO) has been widely used in the treatment of cardiogenic shock (CS) following acute myocardial infarction (6). It provides rescue hemodynamic support to maintain sufficient organ perfusion in refractory CS. The development of percutaneous techniques has made the procedure simpler and safer. Ehrenberger et al. reviewed the current understanding of the VA-ECMO when used in the catheterization laboratory in the setting of CS induced by acute coronary syndrome. They discussed patient selection, timing of implantations, possible complications, and weaning protocol. They also offered their recommendations regarding peripheral VA-ECMO in the catheterization laboratory.

In conclusion, the remarkable advancements in ECLS have continued to revolutionize critical care for patients in dire need. From the selection of patients to early recovery plans, the practice of ECLS remains to be optimized. A lack of clinical guidelines in certain patient population, unclear implications of new mechanical circulatory systems, and changing supporting protocols reflect the urgency for further research. As we move forward, it is essential to focus on refining ECLS techniques, optimizing patient selection, and enhancing its safety. Together, we can continue to explore novel applications, develop innovative devices, and improve the overall quality of care provided to critically ill patients.

## Author contributions

G-wT: Writing – original draft, Writing – review & editing. ND: Writing – original draft, Writing – review & editing. MH: Writing – original draft, Writing – review & editing. ZL: Writing – original draft, Writing – review & editing.

## References

[1] Rabie AA Elhazmi A Azzam MH Abdelbary A Labib A Combes A. Expert consensus statement on venovenous extracorporeal membrane oxygenation ECMO for COVID-19 severe ARDS: an international Delphi study. Ann Intensive Care. (2023) 13:36. 10.1186/s13613-023-01126-9 37129771 PMC10152433

[2] Combes A Price S Slutsky AS Brodie D. Temporary circulatory support for cardiogenic shock. Lancet. (2020) 396:199–212. 10.1016/S0140-6736(20)31047-3 32682486

[3] Ferreira DDC Marcolino MAZ Macagnan FE Plentz RDM Kessler A. Safety and potential benefits of physical therapy in adult patients on extracorporeal membrane oxygenation support: a systematic review. Rev Bras Ter Intensiva. (2019) 31:227–239. 10.5935/0103-507X.20190017 31090853 PMC6649220

[4] Song X Wang H Kashani KB Wang C. Extracorporeal membrane oxygenation using a modified cardiopulmonary bypass system. J Transl Intern Med. (2022) 10:175–177. 10.2478/jtim-2022-0015 35959450 PMC9328033

[5] Su Y Liu K Zheng JL Li X Zhu DM Zhang Y. Hemodynamic monitoring in patients with venoarterial extracorporeal membrane oxygenation. Ann Transl Med. (2020) 8:792. 10.21037/atm.2020.03.186 32647717 PMC7333156

[6] Al-Atta A Zaidan M Abdalwahab A Asswad AG Egred M Zaman A. Mechanical circulatory support in acute myocardial infarction complicated by cardiogenic shock. Rev Cardiovasc Med. (2022) 23:71. 10.31083/j.rcm2302071 35229562

